# Hepatopulmonary Syndrome in a Patient With Autoimmune Hepatitis and Chronic Hepatitis C: A Case Report Highlighting Typical Echo Findings

**DOI:** 10.7759/cureus.41491

**Published:** 2023-07-07

**Authors:** Gagandeep Singh Arora, Hamna Javed, Parneet Kaur, Simran Singh

**Affiliations:** 1 Internal Medicine, University of California Riverside, San Bernardino, USA; 2 Internal Medicine, Saint Agnes Medical Center, Fresno, USA; 3 Internal Medicine, Suburban Community Hospital, Philadelphia, USA; 4 Internal Medicine, Jinnah Sindh Medical University (SMC), Karachi, PAK

**Keywords:** liver transplantation, symptom management, respiratory failure, echocardiography, chronic hepatitis c, autoimmune hepatitis, arterial hypoxemia, intrapulmonary vascular dilatations, hepatopulmonary syndrome

## Abstract

Hepatopulmonary syndrome (HPS) is a rare complication of liver disease characterized by intrapulmonary vascular dilatations leading to arterial hypoxemia. We present the case of a 59-year-old female with a past medical history of bilateral breast cancer status post mastectomy who presented with progressive dyspnea on exertion and fatigue. A comprehensive diagnostic workup was conducted to exclude other cardiac, pulmonary, and systemic etiologies. She was diagnosed with autoimmune hepatitis along with chronic hepatitis C. Echocardiography revealed characteristic findings of intrapulmonary shunting characteristic of HPS. The patient showed improvement in pulmonary symptoms and oxygenation status following the initiation of steroid therapy. Although corticosteroids are not the definitive treatment for HPS, they were considered a supportive measure in this case. However, it is important to note that liver transplantation remains the definitive treatment for HPS. This case underscores the importance of echocardiography and the potential role of supportive measures, like corticosteroids, in managing HPS-related symptoms, particularly in patients with autoimmune hepatitis, as a bridging therapy while awaiting liver transplantation.

## Introduction

Hepatopulmonary syndrome (HPS) is a pulmonary vascular complication occurring in the context of chronic liver disease [1]. It classically presents in cirrhotic patients, but it has also been reported in patients with hypoxic hepatitis and fulminant viral hepatitis [2, 3].

It manifests as intrapulmonary vascular dilatations, resulting in ventilation-perfusion mismatch and arterial hypoxemia [1]. Echocardiography plays a vital role in HPS diagnosis by revealing characteristic findings [4]. Its precise pathophysiology involves complex interactions among liver dysfunction, pulmonary vasculature changes, and impaired oxygenation [1]. Symptoms of HPS include exertional dyspnea, platypnea (shortness of breath when upright), and orthodeoxia (fall in arterial oxygen saturation when upright) [1].

Diagnosis of HPS necessitates the presence of liver disease, intrapulmonary vascular dilatations, and arterial hypoxemia unrelated to other pulmonary or cardiac causes [1]. Echocardiography can identify these features, differentiating HPS from other causes of hypoxemia and guiding appropriate management [4]. 

While liver transplantation is the primary treatment for HPS [5], as it reverses pulmonary abnormalities, various pharmacological treatments like Pentoxifylline, Methylene blue, and Norfloxacin have been attempted as a bridge to transplantation [6]. Though reversion of severe hepatopulmonary syndrome symptoms with steroids has been reported in the past, the role of steroids is not well established [7].

In this case report, we emphasize the importance of recognizing HPS as a potential complication of liver disease. We describe typical echocardiographic findings associated with HPS.

We present a case of HPS in a patient with untreated autoimmune hepatitis and hepatitis C. In our case, echocardiography confirmed the diagnosis and symptom relief was observed after initiating steroid treatment.

## Case presentation

A 59-year-old female with a history of bilateral breast cancer status post-mastectomy, radiation, and chemotherapy almost 10 years ago presented to the emergency department after two weeks of gradually progressive shortness of breath. Her symptoms worsened with exertion and were associated with wheezing. She denied chest pain, palpitations, and dizziness. Besides that patient also complained of right thigh pain and swelling but denied a history of trauma, fever, polyarthralgia, polyarthritis, or abscess in that region.

On physical exam patient's chest was clear to auscultation bilaterally, the only significant finding was right thigh tenderness and swelling. The skin was tender but non-erythematous. She was put on nasal oxygen as she was saturating at 87% on room air, arterial blood gas analysis was done (Table 1) showed PaO2 of 58 mmHg, and A-a gradient was calculated to be 51.73 mmHg.

**Table 1:** Arterial blood gas report on arrival pH: Blood pH, PaO2: Arterial Oxygen Partial Pressure, PaCO2: Arterial Carbon Dioxide Partial Pressure, HCO3-: Bicarbonate Ion Concentration, SaO2: Arterial Oxygen Saturation, FiO2: Fraction of Inspired Oxygen, BE: Base Excess

Parameters	Results	Normal Range
pH	7.42	7.35-7.45
PaO2	58 mmHg	75 - 100 mmHg
PaCO2	32 mmHg	35 - 45 mmHg
HCO3-	24 mEq/L	22 - 28 mEq/L
SaO2	92%	> 95%
FiO2	0.21	Varies based on therapy
BE	-1 mEq/L	-2 to +2 mEq/L

X-ray chest was done which showed no acute cardiopulmonary abnormality. An X-ray of the right lower limb was ordered which ruled out any acute fracture. Blood investigations done in the ER showed that the patient's liver enzymes were elevated, with maximum alanine transaminase (ALT) reaching 408 and aspartate aminotransferase (AST) reaching 1374, thrombocytopenia (77,000 / microliter), and creatine kinase (CK) level was elevated to 18,000 IU/L. Ultrasound abdomen and hepatitis panel were ordered because of elevated liver enzymes. Ultrasound findings demonstrated cholelithiasis without gallbladder wall thickening and a nodular contour of the liver, likely indicating cirrhosis. The diagnosis of cirrhosis was new for her. She tested positive for Hepatitis C. Rhabdomyolysis was thus suspected in the patient's right thigh, as the patient's CK levels were highly elevated. An autoimmune etiology was suspected for the patient's right thigh pain thus antinuclear antibody (ANA) level was ordered which was elevated. 

**Figure 1:**
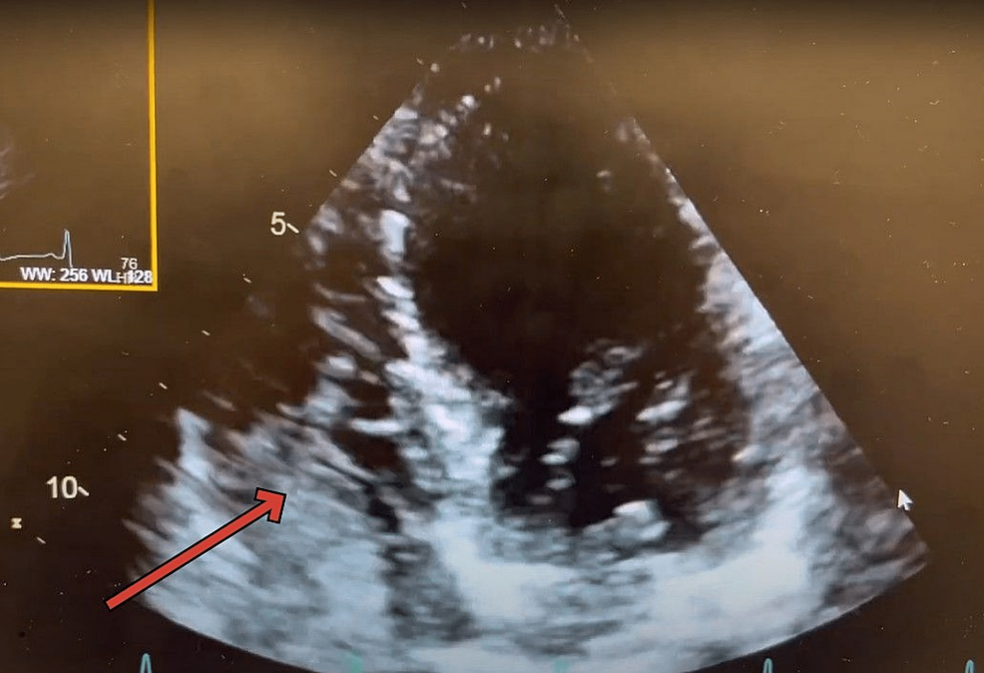
Agitated saline Bubbles are seen entering into the RA as soon as they are injected RA- Right Atrium

**Figure 2:**
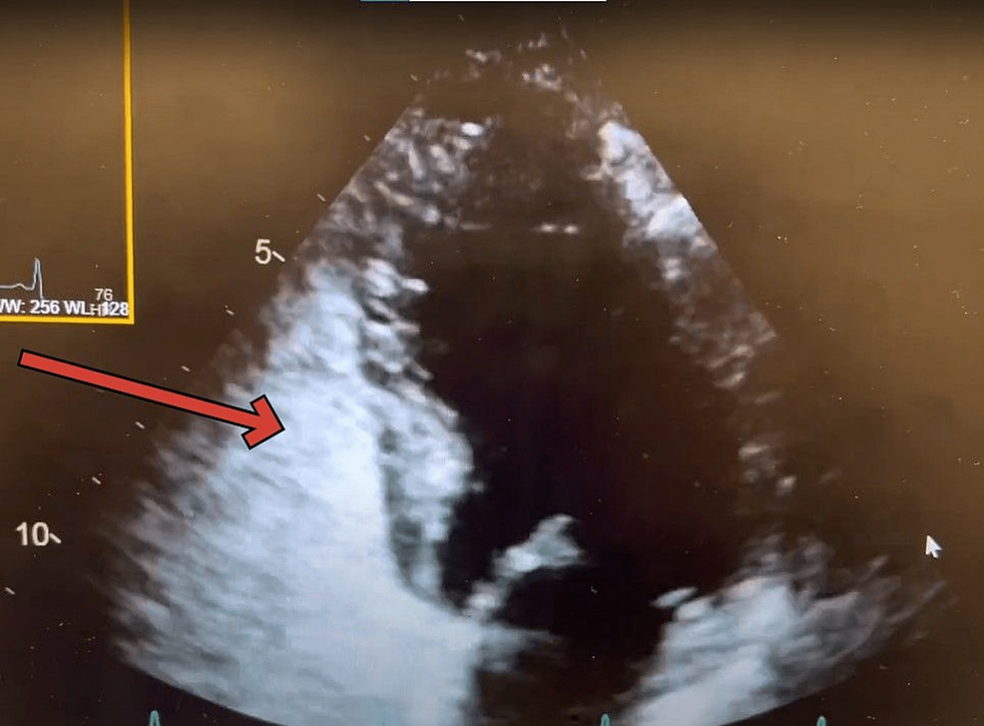
RV seen completely opacified by agitated saline bubbles RV- Right Ventricle

**Figure 3:**
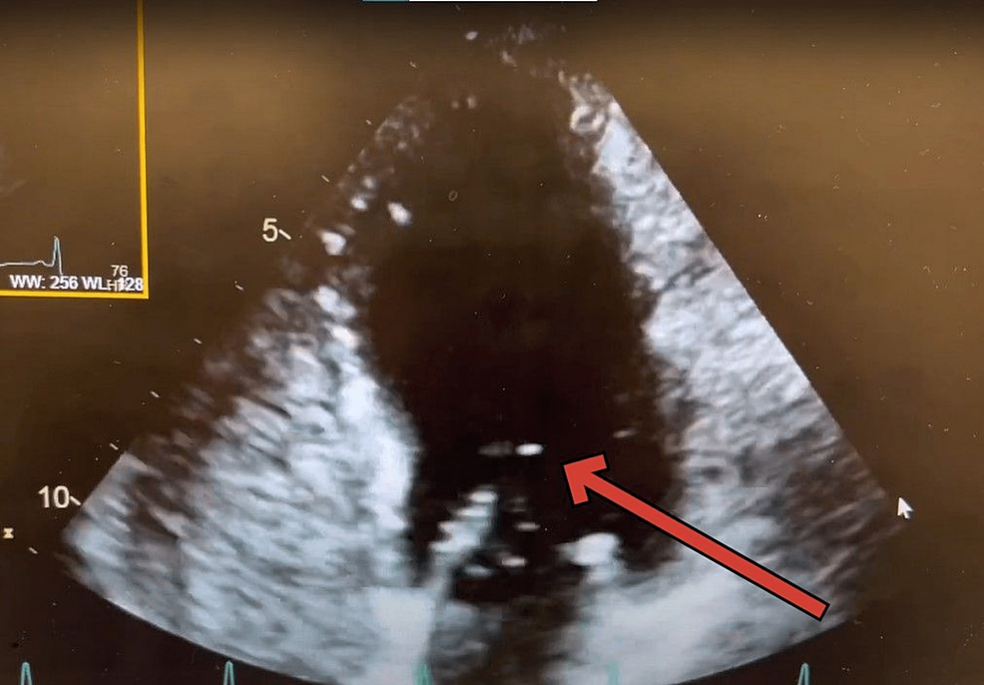
Bubbles seen entering into the LV from LA after 7 cardiac cycles LV- Left ventricle, LA- Left Atrium

Gastrointestinal (GI) suspected hepatopulmonary syndrome and recommended transthoracic echo with bubble and a polymerase chain reaction (PCR) test to quantify hepatitis C virus (HCV) RNA level in the blood. They suspected that the patient may have autoimmune hepatitis considering the patient had a positive ANA with suspicion for polymyositis besides having chronic hepatitis C. HCV RNA level in the blood came out elevated as well 853,114 IU/mL. A transthoracic echo with bubble study revealed mild transpulmonary shunt consistent with hepatopulmonary syndrome (Figures 1, 2, 3) and (Video 1). The echo also showed normal left ventricular wall motion, ejection fraction estimate of 60-65%, normal right ventricle size and function, and a suspicion of a bicuspid aortic valve with normal pulmonary artery pressures.

**Video 1:** Echocardiographic findings typical of Hepatopulmonary pulmonary syndrome

The diagnosis of hepatopulmonary syndrome was thus established based on the typical echocardiographic findings of intrapulmonary shunting, positive saline contrast study, and arterial hypoxemia. Autoimmune antibodies, such as ANA, ANA HEP-2, anti-SSA-52(RO), and anti-smooth muscle antibody with cytoplasmic pattern antibodies came back significantly positive on the autoimmune hepatitis panel. The patient was started on steroids (prednisone 40 mg daily) and a liver biopsy was ordered.

Liver biopsy confirmed dense portal and periportal lymphoplasmacytic inflammation, liver fibrosis (stage 3-4), and no evidence of cholestasis, bile duct proliferation, ductopenia, steatosis, or lesional malignancy. Transfer to a higher level of care for possible liver transplant evaluation was recommended but the patient could not be transferred so an outpatient evaluation for liver transplant was planned. During this period, the patient's liver function tests, CK levels, and creatinine levels improved and returned to baseline. After starting steroids there was a significant improvement in the patient's respiratory status.

The patient was discharged with instruction to continue prednisone 40 Mg for four days followed by 20 mg daily, with follow-up appointments scheduled with a rheumatologist for autoimmune hepatitis and a hepatologist for HCV treatment. 

## Discussion

HPS is a condition characterized by liver disease causing abnormal oxygenation, via intrapulmonary vasodilation [1]. The pathophysiology of HPS involves liver fibrosis leading to increased production of proinflammatory cytokines, such as endothelin 1 and TNF-A, which cause pulmonary dilatation mediated by excessive nitrous oxide production and accumulation of macrophages and monocytes that promote angiogenesis and pulmonary capillary proliferation [1]. An increase in blood flow with preserved alveolar oxygenation causes shunting between the arterial and venous circulation, resulting in a ventilation-perfusion mismatch and arterial hypoxemia [1]. Intrapulmonary vascular dilatations can occur in recognizable patterns, with type 1 lesions having diffuse pulmonary vascular dilatations at the precapillary level and type 2 lesions being more concrete, localized dilatations with large arteriovenous communications distant from the gas exchange [1]. Type 1 lesions respond positively to oxygen therapy, while type 2 lesions have a poor response due to the presence of large true shunts [1]. 

Pulmonary gas exchange abnormality is thus because of the dilation of pulmonary capillary vessels and increasing the number which causes right-to-left shunting, ventilation-perfusion defect, and diffusion limitation [8].

It is diagnosed when an individual with liver disease exhibits a partial pressure of oxygen in arterial blood (PaO2) level of less than 80 mmHg, an A-a gradient corrected for age greater than 15 mmHg (or 20 mmHg if age > 64 years) while breathing room air and intrapulmonary vasodilation confirmed by contrast-enhanced echocardiography or lung perfusion scanning showing a brain shunt fraction greater than 6% [1]. 

Contrast-enhanced transthoracic echocardiography is less invasive and easily available when compared to pulmonary angiography and transesophageal echocardiography [4]. At the same time, it is more sensitive than the technetium 99m-labeled macroaggregated albumin test or Lung perfusion scans [4]. Thus it is the investigation of choice.

Echocardiography plays a crucial role in the diagnosis of HPS, as it can reveal characteristic findings associated with the condition, including intrapulmonary vascular dilatations by evidence of intrapulmonary shunting [4]. By identifying these echocardiographic features, clinicians can differentiate HPS from other causes of hypoxemia and guide appropriate management [4].

The timing of the appearance of the left-sided bubbles after injection can determine the source of the shunt [9]. In intracardiac shunting, the bubbles appear three cardiac cycles after the appearance of the bubbles in the right heart chambers [9]. In intrapulmonary shunting, the bubbles appear four to six cardiac cycles after the appearance of the bubbles in the right heart chambers [9]. 

Various drugs available for treatment include drugs that inhibit nitric oxide synthase (pentoxifylline, methylene blue, L-NG-nitro arginine methyl ester, quercetin, mycophenolate mofetil,caffeic acid phenethyl ester, N- acetylcysteine), drugs that inactivate endothelial-1 (pentoxifylline, quercetin, mycophenolate mofetil), drugs that inhibit pulmonary angiogenesis (pentoxifylline, quercetin, and mycophenolate mofetil) and drugs that inhibit bacterial translocation and cause subsequent decrease in nitric oxide (norfloxacin) [6].

Tzovaras et al. in 2006 reported a case where they observed complete reversion of hepatopulmonary syndrome (HPS) following corticosteroid treatment in a non-cirrhotic patient with portal hypertension due to idiopathic granulomatous hepatitis. This finding highlights the unique response to corticosteroids in this patient and expands our understanding of HPS management at least in the context of granulomatous hepatitis [7].

In our case, a Liver biopsy proved the patient had cirrhosis and the patient observed improvement of symptoms with supportive therapy and steroid use. The patient had Autoimmune hepatitis and chronic hepatitis C. The use of steroids in the management of hepatopulmonary syndrome symptoms in cirrhotic and autoimmune hepatitis patients needs further research. 

The presence of HPS is considered an exception to the model for end-stage liver disease (MELD) score and patients are given priority for liver transplantation [10].

## Conclusions

In conclusion, the case highlights the diagnostic significance of echocardiography in confirming hepatopulmonary syndrome in a patient with untreated hepatitis C and autoimmune hepatitis. The characteristic echocardiographic findings of intrapulmonary vascular dilatations, intrapulmonary shunting, and arterial hypoxemia were instrumental in establishing the diagnosis. While corticosteroids are not established as a primary treatment for hepatopulmonary syndrome, their use in this particular case may have been beneficial due to the presence of autoimmune hepatitis. The patient demonstrated improvement in pulmonary symptoms and oxygenation status with supportive corticosteroid therapy. Further research is warranted to explore the potential role of corticosteroids in managing hepatopulmonary syndrome associated with autoimmune hepatitis.
